# The impact of rural hospital closures on equity of commuting time for haemodialysis patients: simulation analysis using the capacity-distance model

**DOI:** 10.1186/1476-072X-11-28

**Published:** 2012-07-23

**Authors:** Masatoshi Matsumoto, Takahiko Ogawa, Saori Kashima, Keisuke Takeuchi

**Affiliations:** 1 Department of Community-Based Medical System, Faculty of Medicine, Hiroshima University, 1-2-3 Kasumii, Minami-ku, Hiroshima 734-8551, Japan; 2 Centre for Kidney Diseases, Hiroshima Prefectural Hospital, 1-5-54 Ujina-kanda, Minami-ku, Hiroshima 734-8530, Japan; 3 Department of Public Health and Health Policy, Hiroshima University Institute of Biomedical & Health Sciences, 1-2-3 Kasumii, Minami-ku, Hiroshima 734-8551, Japan

**Keywords:** Renal dialysis, Geographic information systems, Rural health, Computer simulation, Transportation, Japan

## Abstract

**Background:**

Frequent and long-term commuting is a requirement for dialysis patients. Accessibility thus affects their quality of lives. In this paper, a new model for accessibility measurement is proposed in which both geographic distance and facility capacity are taken into account. Simulation of closure of rural facilities and that of capacity transfer between urban and rural facilities are conducted to evaluate the impacts of these phenomena on equity of accessibility among dialysis patients.

**Methods:**

Post code information as of August 2011 of all the 7,374 patients certified by municipalities of Hiroshima prefecture as having first or third grade renal disability were collected. Information on post code and the maximum number of outpatients (capacity) of all the 98 dialysis facilities were also collected. Using geographic information systems, patient commuting times were calculated in two models: one that takes into account road distance (distance model), and the other that takes into account both the road distance and facility capacity (capacity-distance model). Simulations of closures of rural and urban facilities were then conducted.

**Results:**

The median commuting time among rural patients was more than twice as long as that among urban patients (15 versus 7 minutes, p < 0.001). In the capacity-distance model 36.1% of patients commuted to the facilities which were different from the facilities in the distance model, creating a substantial gap of commuting time between the two models. In the simulation, when five rural public facilitiess were closed, Gini coefficient of commuting times among the patients increased by 16%, indicating a substantial worsening of equity, and the number of patients with commuting times longer than 90 minutes increased by 72 times. In contrast, closure of four urban public facilities with similar capacities did not affect these values.

**Conclusions:**

Closures of dialysis facilities in rural areas have a substantially larger impact on equity of commuting times among dialysis patients than closures of urban facilities. The accessibility simulations using thecapacity-distance model will provide an analytic framework upon which rational resource distribution policies might be planned.

## Background

The importance of access to healthcare facilities depends on the kind and severity of disease a patient has. For sustaining their lives, patients undergoing haemodialysis are required to commute to dialysis facilities three times a week. Accessibility to these facilities has an important impact on dialysis patients much more than patients with other chronic conditions. Access is determined by various factors such as geographic accessibility, economic affordability, availability of facility, and the patient’s preference [1,2]. What factors are relatively more important than others are also dependent on the disease. Poor geographic accessibility, represented as long commuting distance, increases mortality rate among dialysis patients [3-5]. The frequent and long-term commuting leads to a huge cumulative time and become a burden on the patients’ lives. Different from other treatments, the capacity of a dialysis facility is strictly determined by the number of dialysis consoles and the workforce at the facility. Thus, a patient cannot necessarily be treated by the nearest dialysis facility. Access for dialysis patients thus depends largely on geographic accessibility and facility availability.

Because dialysis costs more than treatment modalities for other chronic diseases, economic status of a patient can influence the access in some countries [6,7]. In Japan and some other welfare states, however, self-payment for dialysis treatment is very small, which means financial accessibility is not terribly important to the patients. In Japan, the entire population is covered by social health insurance which is financially supported by the government [8]. The price of each medical service is strictly determined by the government and thus is generally lower than the price in market-dependent health systems such as those in the United States [8,9]. In addition to the universal health insurance coverage and lower cost of services, there is special financial support to dialysis patients from public expenses, which makes it possible for a remarkably large number of patients with renal diseases to have access to dialysis. The number of dialysis patients in Japan in 2007 was 215 per 100,000 people, which was much larger than the 122 in the US and 40 in Europe [10]. The rate of kidney transplantations among end-stage renal disease (ESRD) patients, however, is much lower in Japan (0.3%) than in the US (26%) and UK (56%) [11,12].

Geographic accessibility, however, is an issue in Japan and there is a substantial gap in this aspect of access. Private medical institutions provide the majority of medical care in Japan; 82% of hospitals and 96% of clinics are in the private sector [13]. The national and local governments have no right to politically intervene on locations of these private institutions. Thus, similar to many other countries, distribution of healthcare resources is highly concentrated in urban areas [14,15]. It is expected that there is a substantial gap in patient accessibility to dialysis facilities. What is worse, since 2004, when the new postgraduate training scheme was implemented, the existing urban–rural imbalance of physician distribution has been deteriorating [16-18]. Rural hospitals, especially rural public hospitals with low profitability are now undergoing privatisations, downsizings, mergers, and closures at an ever-accelerating rate [19,20]. These phenomena potentially and unfavorably impact the geographic accessibility of some patients. The closures of rural hospitals are also reported as a social problem in other countries [21-24].

Because access is a direct matter of life, it is politically important to study the equity in accessibility among dialysis patients and predict the impact of facility closures on the equity. For this purpose, simulation analysis of access using geographic information systems (GIS) is indispensable. Conventional models simulating access of dialysis patients take only geographic accessibility into account (i.e., the models assume that a patient commutes to the nearest facility in linear or road distance) [4,25-27]. These models are likely to overestimate patient accessibility. Moreover, these models cannot precisely simulate closures of or capacity transfer among facilities. A new model is needed that incorporates both geographic accessibility and facility capacity [28-36]. Location-allocation model is a method for deciding locations for facilities and simultaneously assigning spatially-distributed sets of demands to the facilities [29,37]. The location-allocation model can be further optimised by adding maximum and/or minimum capacity of each facility to the model [29,38]. The model incorporating facility capacity can simulate access of users in a more realistic manner than the model without facility capacity [28]. Among various types of healthcare facilities, the capacity-included location-allocation model is often applied to primary care and preventive service facilities, both of which, like dialysis facilities, are suited more to decentralisation than centralization [28,32,35,36,38]. A trial of applying this model to dialysis facilities, however, has scarcely been conducted.

In this study, we evaluate the equity of commuting times of dialysis patients in Hiroshima prefecture, Japan, using the new model embedded in GIS in which travel time and facility capacity are both taken into consideration. Simulations are conducted to examine the impact of closures of some rural dialysis facilities on the equity of commuting times, in comparison with the impact of closures of urban facilities with similar capacities. Also, the impact of capacity transfer between urban and rural facilities was evaluated. Through these analyses, we show the gap of results between the conventional and the new model, and discuss the advantages of using the new model for planning policies for health resource allocation.

## Materials and methods

### Study area and subjects

Targeted subjects are all the 7,374 persons that are certified as the “renal disabled” in the Hiroshima prefecture. The status of “renal disabled” is certified by a municipal government in which the person lives based on the report from a physician that cares for the person. With financial support from public expenses, the copayment for dialysis therapy of a certified “renal disabled” patient is totally exempted or is reduced from 20,000 to 10,000 yen per month depending on the household income. The information on the certified disabled are registered by each municipality government. There are three types of “renal disabled”: first, third and fourth grade. To be certified as first grade disability, the serum creatinine level of the person is required to be no less than 8.0 mg/dl or creatinine clearance to be less than 10 ml/min. For third grade, serum creatinine level must be 5.0-8.0 mg/dl or creatinine clearance 10–20 ml/min. For fourth grade, the serum creatinine level must be 3.0-5.0 mg/dl or creatinine clearance 20–30 ml/min [39]. For this study, the post code information as of August 1, 2011 of the entire first and third grade “renal disabled” patients were collected from all the 23 municipalities of Hiroshima prefecture (capture rate 100%).

Because of the copayment alleviation for dialysis therapy and various non-medical benefits, those with renal impairment usually apply for certification of first or third grade renal disability when they start dialysis. As a preliminary survey, we collected information on certified disability status of all the dialysis patients as of June 2011 from seven medical institutions (three in Hiroshima city and four in surrounding rural areas). Among the 486 dialysis patients at the institutions, 483 (99.3%) were certified as having first or third grade renal disability.

Information on postal address, the number of dialysis consoles, and the maximum number of outpatients (capacity) of each dialysis facility were collected from 90 facilities on the list of members of the Japanese Society for Dialysis Therapy. Eight facilities that were not on the list were contacted directly by authors. The capacity of each facility was set as the maximum number of commuting patients that the director of the facility considered the facility capable of accepting based on, for example, number of consoles and availability of human resources.

### Calculation of commuting time using GIS

**Figure 1:**
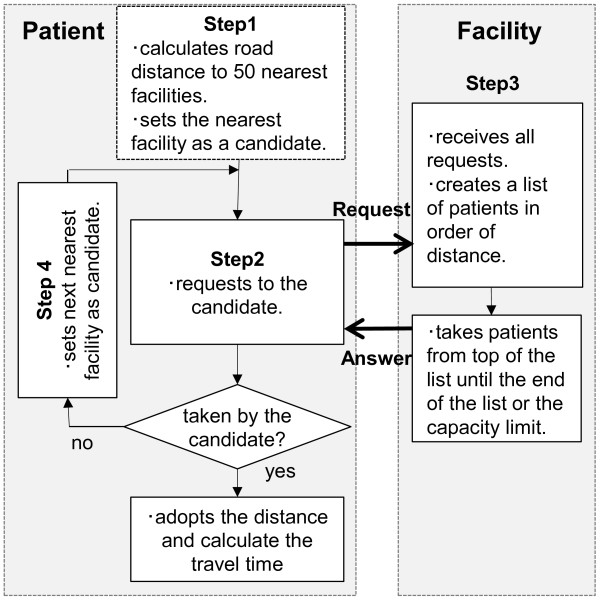
Algorithm determining the commuting facility and road distance for a patient in the capacity-distance model.

First, patients and facilities were geocoded according to their addresses. Next, travel times by car were calculated based on two models: distance model and capacity-distance model. The distance model does not take into account the capacity of each facility. In this model, among all the facilities, the facility with the shortest travel time was regarded as the place a patient would choose. In the capacity-distance model, the capacity of each facility was incorporated into the calculation algorithm (Figure 1). First, each facility accepted patients in order of shorter travel time until it reached the limit of its capacity. Second, if a patient was not accepted by the facility in the first step, the patient approached the next-nearest facility in the same manner as the first step. Then, it ran through the first and second steps until all the patients were accepted by any one of the facilities.

In the calculation process, we carried out network analysis (i.e., discerned the shortest travel-path between two locations on a road network including highways), to find the travel time (minutes) by car from each patient’s home to each dialysis facility. The travel time was calculated using GIS software ArcGIS version 10.0 (ESRI Japan Inc.) and ArcGIS Data Collection Road Network 2011(ESRI Japan Inc.). In the Road Network, driving speeds of all the segments of the roads are classified into 14 categories depending on the type and width of the segment.

### Closure and transfer simulations

**Figure 2:**
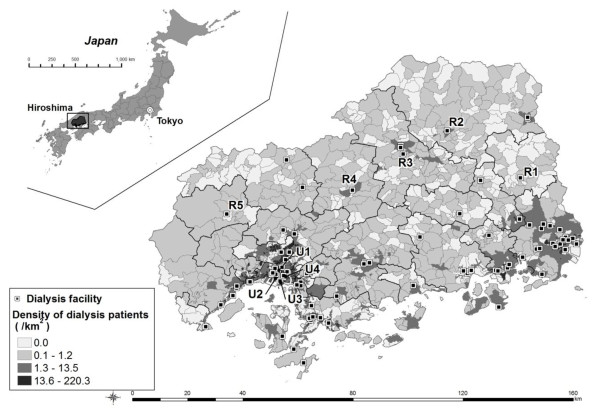
**Distribution of dialysis patients and facilities.** Footnote: U1-4 and R1-5 are target urban and rural hospitals in closure simulations.

Simulations of closures of rural facilities and inter-facility capacity transfers were conducted. Five rural public hospitals and four urban public hospitals were chosen as the targets of the simulations (R1-5 and U1-4, shown in Figure 2). Definitions of “urban” and “rural” areas are described later. In Japan, 80% of maintenance haemodialysis is provided by private facilities [11]. Because these private facilities are out of reach of political intervention, they were excluded from the targets of closure, but remained in the model. The five rural public hospitals (R1-5) selected were, among all public facilities in rural areas, certified by the prefectural government as “rural health hub hospitals”, whose managements are politically focused and subject to intervention by the government. The capacity of R1-5 was 15, 70, 100, 116 and 18, respectively, while the median (range) of capacity of all the 22 rural facilities was 34 (5140). All the five hospitals are suffering from a shortage of doctors; the number of doctors has substantially decreased for these ten years in all the hospitals. As the control group of the rural hospitals, all the four public hospitals (U1-4) in the old districts of Hiroshima City (the most densely populated areas of the capital city of the prefecture) were selected. The capacity of U1-4 was 90, 124, 80 and 30, while the median (range) of capacity of all the 76 urban facilities was 76 (2640). The total capacity of the four urban hospitals (324) and that of five rural hospitals (319) were almost equal and each consists of 3.7% of the total capacity of all the facilities (8,643) in Hiroshima.

First, in the simulations, each of the rural hospitals was closed by setting its capacity at zero. Then, all the five rural hospitals were closed. In a similar manner, all the four urban hospitals were closed. Finally, the capacity of the four urban hospitals was transferred to the five rural hospitals by closing the urban hospitals and doubling the capacity of the rural hospitals.

### Geographic unit and statistical analyses

The second-smallest census block was employed as the geographic unit for analysis. The census block is smaller than a municipality (city, town, or village), which is the basic administrative unit of Japan. Hiroshima prefecture has 23 municipalities which consist of 1,867 census blocks. The census blocks were sorted according to population density and divided into urban and rural areas so that 25% of patients in the blocks with the lowest population density were included in the rural area. The cut-off point in population density was 770 per square kilometer.

The difference in commuting times for patients between urban and rural areas was examined using the Mann–Whitney test. Equity of commuting time among patients was evaluated with Gini coefficient. All of the patients were ranked by commuting time, and the cumulative proportion of commuting time and that of individual patients was plotted onto the plane of coordinates. The plotted line is the Lorenz curve, and the Gini coefficient is the area between the Lorenz curve and the 45^o^ line which is divided by the triangle under the 45^o^ line. Gini coefficient varies between 0 (complete equity) and 1 (complete inequity) according to the degree of variation in commuting times. All of these statistical analyses were conducted using SPSS version 19 (IBM-SPSS Japan, Tokyo).

The Ethics Committee for Epidemiological Research, Hiroshima University, and The Research Ethics Committee in Hiroshima Prefectural Hospital have assessed and permitted this study.

## Results

Total population of Hiroshima prefecture was 2,876,642 and its area was 8,290 square kilometers. All the 7,374 patients with certification of “renal disabled” and 98 dialysis facilities were registered in this study. The total number of dialysis consoles was 2,381 and the total capacity of the facilities was 8,643. The medians (interquartile ranges) of population, area and the number of patients in 1,876 census blocks were 782 (2901,843), 1.0 (0.2-5.6) square kilometers, and 2 (05), respectively. The values in 870 rural blocks were 375 (164915), 5.9 (2.7-11.5), and 1 (02). The values in 997 urban blocks were 1,329 (6442,877), 0.3 (0.1-0.6), and 3 (18). Geographic distribution of patients and facilities are shown in Figure 2.

**Figure 3:**
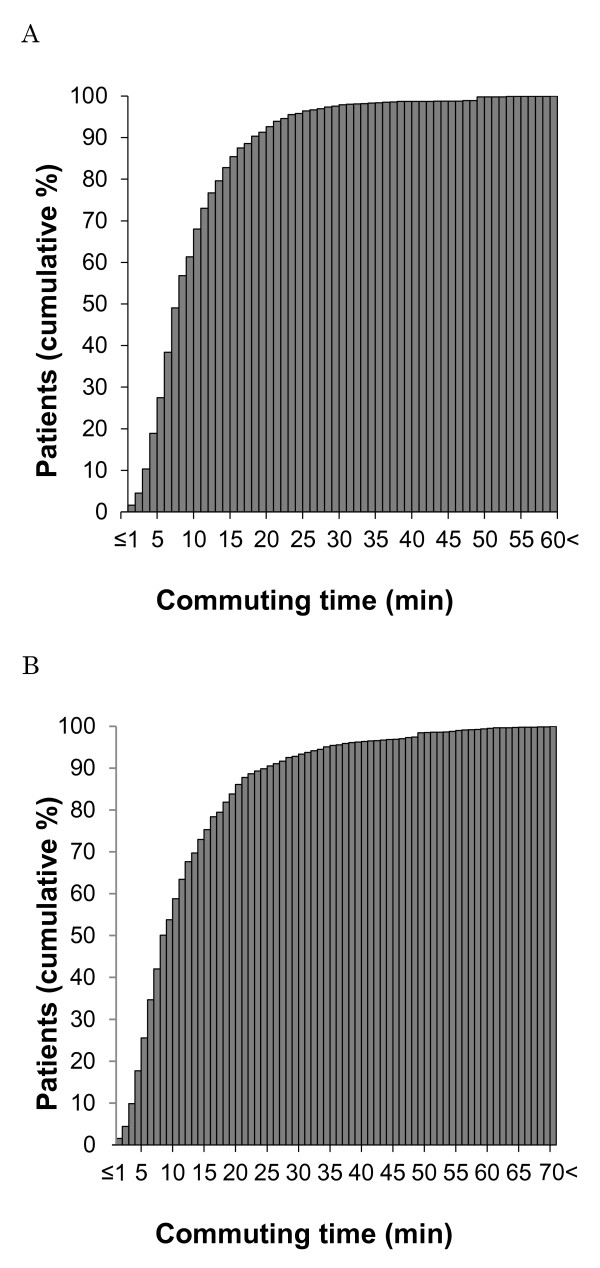
Distribution of dialysis patients according to commuting time (A: Distance model; B: Capacity-distance model).

Results of commuting time calculations are shown in Table 1. In the distance model, commuting times for dialysis patients varied from 0 to 60 minutes and its median was 7 minutes in the whole prefecture. In the capacity-distance model, commuting times were prolonged compared with the distance model; the range was between 0 and 96 minutes and the median was 8 minutes. In the capacity-distance model, the median commuting time among rural patients was more than twice as long as that among urban patients (15 versus 7 minutes, p < 0.001). Gini coefficient of commuting times of urban patients was slightly lower than that of rural patients (0.389 versus 0.405 in the capacity-distance model), indicating slightly better equity among urban patients than rural patients. Taking all the patients into account, the Gini coefficient was even larger (0.438 in the capacity-distance model), reflecting the gap in commuting time between urban and rural areas. Equity of commuting time among patients in each model is also shown in Figure 3.

**Table 1:** Commuting time of dialysis patients

	**Whole prefecture**		**Urban**		**Rural**		P (urban vs rural)*	
	Model**		Model**		Model**		Model**	
	**1**	**2**	**1**	**2**	**1**	**2**	**1**	**2**
N	7374	7374	5538	5538	1836	1836		
Median (min)	7.09	7.97	6.32	6.94	13.26	15.26	<0.001	<0.001
IQR (min)	4.92-11.48	4.98-14.90	4.43-9.25	4.60-11.21	7.89-20.12	9.37-26.32		
Range (min)								
Minimum	0	0	0	0	0	0		
Maximum	59.63	96.03	35.86	60.24	59.63	96.03		
Gini coefficient	0.381	0.438	0.311	0.389	0.363	0.405		

*Mann -Whitney test.

**Model 1: distance model; Model 2: capacity-distance model.

IQR: interquartile range.

Discrepancy in commuting facilities and gap of commuting times between the distance and the capacity-distance models are shown in Table 2. Of all the patients, 2,662 (36.1%) commuted to the facilities that were not the nearest (facility rank 2 or lower in Table 2); that is, they commuted to different facilities in the two models. The lower the facility rank is in the capacity-distance model, the gap of commuting time between the two models tended to be larger. Patients that had the largest gap commuted to the 36^th^-nearest facility in the capacity-distance model and the gap of commuting time was 60 minutes.

**Table 2:** Gap of commuting time between the distance and the capacity-distance model (sorted according to the proximity rank of commuting facility in the capacity-distance model)

		**Commuting time (min)**	
Rank*	N**	**Median**	**IQR**
1	4712	0.00	0.00 - 0.00
2	1019	1.23	0.56 - 2.31
3	445	2.39	1.50 - 4.73
4	353	5.81	4.12 - 7.23
5	310	6.90	5.28 - 11.01
6	124	5.46	5.30 - 6.08
7	33	8.78	5.49 - 8-78
8	14	10.47	10.47 - 10.49
9	25	26.56	7.50 - 26.56
10	40	8.97	5.46 - 13.67
11	18	19.13	10.47 - 29.18
12	39	21.63	6.95 - 26.25
13	55	13.87	13.27 - 20.15
14	28	12.31	12.31 - 25.56
15	46	25.47	13.60 - 26.11
16	51	21.99	20.87 - 21.99
17	22	12.31	12.05 - 33.17
18	3	33.58	12.31 - 33.58
19	8	16.47	16.47 - 16.47
21	16	21.99	21.99 - 21.99
23	8	21.99	19.49 - 21.99
29	1	58.26	58.26 - 58.26
36	4	60.00	60.00

*Rank of the commuting facility of a patient in from nearest (rank 1) in the capacity-distance model.Rank 2 means the patient commutes to the second-nearest facility in the model, while in the distance model, all patients commute to rank 1 facilities.

**Number of patients.

The patient load at each facility was also different between the models. In the distance model, 47 facilities (total capacity 2,335) accepted the number of patients that exceeded the capacity, which did not happen in the capacity-distance model. The median, range, and total of the number of excess patients at these facilities were 41, 3196, and 2,662, respectively. Also in the distance model, the number of patients at 51 facilities (total capacity 6,308) was below the capacity. The median, range, and total of the excess capacity at these facilities were 45, 4640, and 3,931, respectively.

Results of closure and transfer simulations in the capacity-distance model are shown in Table 3. Closure of any one of the five rural hospitals increased the Gini coefficient and the number of patients with a longer commuting time. When all the five rural hospitals were closed, Gini coefficient increased by 16%; the number of patients with commuting times longer than 60 minutes increased by 7-times, and the number of patients with commuting times longer than 90 minutes increased by 72-times. In contrast, closure of all the four urban hospitals did not affect Gini coefficient significantly, and also did not change the number of patients with long commuting times. When the capacity of the four urban hospitals was transferred to the five rural hospitals, Gini coefficient and the number of long-time commuting patients decreased only slightly.

**Table 3:** Effects of closure and capacity transfer simulations in the capacity-distance model

				**Number of patients**			
	Capacity*	Gini**		**Commuting time (min)**			
			All	30<	45<	60<	90<
No closure		0.4	7374	490	229	39	1
Closure simulations							
Closed hospital(s)							
R1	−15	0.440	7374	505	242	40	1
R2	−70	0.459	7374	537	285	90	31
R3	−100	0.462	7374	569	298	105	11
R4	−116	0.457	7374	577	230	39	4
R5	−18	0.440	7374	507	230	39	4
R1-5	−319	0.507	7374	774	494	256	72
U1-4	−324	0.433	7374	490	229	39	1
Capacity transfer simulation							
Double R1-5 and close U1-4	+319324	0.428	7374	473	206	30	0

*Maximum number of patients of hospital(s) closed or transferred.

**Gini coefficient of commuting time of all the patients.

## Discussion

The results demonstrated that there was a substantial inequity in commuting times of dialysis patients in Hiroshima prefecture. Patients in rural areas had a longer commuting time than urban patients had. Simulation analyses revealed that if public hospitals in rural areas were closed, the equity of commuting times among patients worsened much more than if urban public hospitals of similar capacity were closed. The equity did not change when the capacity of the urban hospitals was transferred to the rural hospitals. The calculated commuting time of each patient and the equity of the commuting among the patients in the new capacity-distance model were substantially different from those in the conventional distance model. In the conventional model, about a half of the facilities accepted a patient load that exceeded capacity.

There are past reports that suggested the usefulness of GIS as a tool for calculating commuting time of dialysis patients [4,25-27,40,41]. In addition to location information such as post code or residential address that have been used in conventional studies [4,25,26], in our study, information on the capacity of each facility was taken into account so that the closest available facility could be identified. This enables us to calculate commuting time which is closer to the real commuting time than the conventional model [28]. The difference between the conventional model and the new model was reflected in difference in results between the two models in this study. Gini coefficient of commuting time in the whole prefecture was 0.38 in the conventional model and was 0.44 in the new model. Commuting times of more than one third of patients differed between the two models. Moreover, with the capacity-distance model, researchers can simulate the effects of closure, opening, and capacity transfer of dialysis facilities in a more realistic manner than they do in the conventional model. In this study, we simulated closure of rural facilities. It is possible to simulate opening of new facilities in rural areas in the same model. We did not conduct the opening simulation because it is currently unrealistic in Japan due to a shortage of financial and human resource in rural areas. Although it is not so strictly determined as in a dialysis facility, the limit of capacity at each medical facility of any kind of treatment does exist. The capacity-distance a model might thus be applied to other diseases that need continuous commuting in other regions of the world.

Due to the start of the new postgraduate training scheme in 2004 and massive mergers of municipalities since 2005, urban–rural imbalance of physician distribution has recently worsened in Japan [16,17]. Most of the rural public facilities in this study are suffering from a shortage of doctors. For example, hospital R1 and R5 shown in Figure 2 experienced a 30% decrease in the number of physicians over the last 10 years. Because of the worsening shortage of physicians, public hospitals—particularly those in rural areas—are under political pressure to re-structure (i.e., close, merge, or privatise) [19,20]. Policy-makers need to understand that such re-organisation can potentially incur great inconvenience for dialysis patients in rural areas. The simulations in this study revealed that even closure of the smallest rural hospital (R1: capacity 15) could affect the equity of commuting times to a greater extent than closure of all the four large urban hospitals (U1-4: total capacity 324).

For certain acute medical care needs, concentration of medical resources at a small number of facilities reportedly improves patient outcomes [42]; that is, there is a trade-off between overall health and resource equity. In these cases, which might include the beginning of dialysis therapy, geographic misdistribution of medical resources might be permitted for the greater benefit of the majority of patients [43]. In the case of maintenance dialysis, however, there is no study that suggests such a trade-off. Rather, patients with longer commuting times have a higher mortality [3,4]. Moreover, because the rate of kidney transplantations is low in Japan, most of the patients with end-stage renal diseases cannot avoid commuting three times per week to dialysis facilities for the rest of their lives. Thus, from the perspective of patient quality of life and ethics, a fair equity in accessibility for patients should be guaranteed. Under the existing misdistribution of facilities and unequal accessibility among patients in Hiroshima prefecture, further and artificial widening of the accessibility gap between urban and rural patients is undesirable.

It is therefore politically recommended that closures and mergers of rural hospitals, which are in process throughout Japan, should be planned very cautiously. Transfer of hospital capacity from urban to rural areas has a very limited beneficial effect on equity of patient accessibility. In addition, these urban hospitals accept many patients throughout the prefecture to start dialysis and also play a central role in caring for patients with multiple complications. Thus, such a transfer is not recommended.

Minimising patient travel distance fits with the current sustainability and equality agenda [44]. Greenhouse gas (GHG) emission attributable to health care provision is substantial; it consists of 7% of total GHG emission in the US and 3% in the UK [45,46]. The shortening of commuting times reduces not only travel cost for dialysis patients, but also GHG emission related to their commuting [45,47]. Also, reducing the number of consoles and cutting down facility capacity reduces GHG emission [47]. If the capacity of each facility is changed so that all the patients can access their nearest facilities and the capacity of all the facilities is completely filled by the patients, the dialysis provision system minimises burdens both for environment and for patients. The results of this study showed that in order to achieve such an optimal system, 52% of the facilities needed to reduce their total capacity by 62% (the total capacity being 0.38-times its original size) and 48% of facilities needed to increase their total capacity by 114% (2.14 times).

In this study, the “renal disabled” were used as the study subjects, but the “renal disabled” are not necessarily identical to dialysis patients. As mentioned in the methods, the preliminary survey demonstrated that almost all the dialysis patients were certified as “renal disabled”. However, it is unknown how great a proportion of all the “renal disabled” are undergoing dialysis. A necessary condition for being certified as first or third grade renal disability is serum creatinine value more than 5 mg/dl. Even if some of the disabled do not receive dialysis now, they will likely require renal replacement therapy in the near future. And even if some non-disabled patients receive dialysis, the number would be very small because they are those rare patients whose serum creatinine values are less than 5.0 mg/dl, but have a very limited response to diuretics or need extra corporeal ultra-filtration methods to avoid pulmonary congestion. In 1991, the average serum creatinine value at the beginning of dialysis in Japan was 10.6, and the value has rapidly dropped to 8.3 mg/dl in 2006, which suggests the value should now be even lower [48]. Moreover, according to the annual report of the Japanese Society for Dialysis Therapy, the number of patients receiving dialysis in 2010 was 7,132 in Hiroshima [49], which is close to the value in this study: 7,374. Considering the fact that the coverage rate of the Society’s survey for its member facilities was 98%, there are dialysis facilities without membership to the Society, and that there is a natural increase in the number of dialysis patients between 2010 and 2011, the real number of patients would be even closer to 7,374. The group of subjects of this study would thus be identical to the population of real patients to a significant degree.

The study subjects included patients receiving peritoneal dialysis, which requires further attention. Although the precise number is unknown from our data, the annual report of the Japanese Society for Dialysis Therapy showed that 6.8% of all the dialysis patients in Hiroshima were undergoing peritoneal dialysis [49]. Because patients with peritoneal dialysis don’t need to commute to facilities as often as patients with haemodialysis, the inequity of accessibility among them may not be so serious an issue than that among haemodialysis patients. Another limitation of this study is that the data and analysis are contained in Hiroshima prefecture only; the results therefore can be applied to this limited region of Japan. The methodology of this study and its applicability, however, are not limited. The closure and transfer simulations in the GIS-embedded capacity-distance model can be used for analysis in other countries and can contribute to making policies on health resource distribution. Also, transportation measures used by patients may be different between urban and rural areas. Generally speaking, public transportation is more available in urban areas than in rural areas. Thus the accessibility of rural patients shown in this study might be overestimated compared with real accessibility.

Finally, the estimated travel time is not identical to actual travel time. In the capacity-distance model, a patient always sends a request to the nearest available facility. In reality, however, a patient would choose a facility based not only on availability and distance, but also on other factors such as means of transportation, working status, level of family support and quality/status of the facility. Also, unlike in the model, a real facility does not necessarily accept patients according to their proximities to the facility. A facility may accept a patient based, for example, on the stage of renal failure and level of complications the patients has. Thus, there may be a gap between the calculated equity of accessibility in this study and the real equity, which is more than just the equity of travel distance.

## Conclusions

This study revealed the substantial impact of closures of dialysis facilities in rural areas, which are now imminent in Japan, on equity of commuting times among dialysis patients. Policies to prevent such closures are recommended. Simulations by the capacity-distance model embedded in GIS will provide evidence upon which rational policies might be planned not only in Japan, but also in other countries, and not only for haemodialysis, but also for other treatment modalities that require repeated, continuous patient commuting.

## Abbreviations

ESRD: End-stage renal disease; GIS: Geographic information systems.

## Competing interests

None

## Authors' contributions

All authors have made contributions to conception and design, or acquisition of data and interpretation of data. MM and SK have conducted analysis. All authors have been involved in drafting the manuscript. All authors have given final approval of the version to be published.
